# AI Chatbots as a source of Ramadan medication management advice for patients with diabetes: a multilingual comparative evaluation

**DOI:** 10.3389/fmed.2026.1934432

**Published:** 2026-08-28

**Authors:** Sufyan Alomair, Maryam Alsuwayq, Walaa Alabbad, Khawlah Albeladi, Zainab Alhassan

**Affiliations:** Department of Pharmacy Practice, College of Clinical Pharmacy, King Faisal University, Al-Ahsaa, Saudi Arabia

**Keywords:** artificial intelligence, ChatGPT, Copilot, diabetes mellitus, Gemini, medication safety, Ramadan

## Abstract

**Background:**

Patients with diabetes increasingly consult artificial intelligence (AI) chatbots for medical advice, including guidance on antidiabetic medication management during Ramadan fasting, because AI can simplify and summarize long, complex guidelines. Also, in hospital settings, these tools are being used in hospitals much faster than it takes to establish formal regulations and guidelines for their use. Evaluations of the accuracy, completeness, and reproducibility of such advice across languages are still lacking. Therefore, the study aims to evaluate and compare the accuracy, completeness, safety, and reproducibility of three widely used AI chatbots—ChatGPT, Google Gemini, and Microsoft Copilot—when providing antidiabetic medication adjustment advice during Ramadan in both English and Arabic.

**Methods:**

Twenty-three standardized clinical scenarios covering common antidiabetic regimens were presented to each chatbot in both English and Arabic. Each query was repeated to evaluate reproducibility, resulting in 276 responses scored. Responses were assessed against the International Diabetes Federation–Diabetes and Ramadan (IDF-DAR) Guidelines using a 0–2 accuracy scale, a 0–4 completeness scale, and a 0–3 safety scale.

**Results:**

Overall, 77% of responses were fully consistent with the guideline, 12% were partially consistent, and 11% (30/276) contained clinically harmful or contradictory advice; harmful responses were about twice as common in Arabic as in English (14% vs. 8%). Completeness and safety were high, with medians at the observed ceiling. In the generalized linear mixed models, chatbots did not differ significantly in accuracy, completeness, or safety, and there was no significant main effect of language or chatbot × language interaction; the strongest signals were a chatbot effect on completeness (*p* = 0.068) and a language effect on safety (*p* = 0.064), both non-significant. Two-week reproducibility was fair for accuracy (weighted κ = 0.20, *p* = 0.009) and completeness (κ = 0.29, *p* = 0.001) and showed a very low κ in the safety scale (κ = 0.02, *p* = 0.81).

**Conclusions:**

AI chatbots demonstrated comparable performance in delivering guideline-based advice for diabetes management during Ramadan, with no significant differences in accuracy, completeness, or safety. While most responses aligned with the IDF-DAR guideline, some harmful recommendations persisted, and response consistency fluctuated over time. These results suggest that AI chatbots should serve as a supplementary resource rather than a substitute for professional medical advice.

## 1 Introduction

Ramadan, as one of the five pillars of Islam, is the 9th month of the Islamic calendar, during which healthy adults abstain from food, fluids, and medications from dawn (Suhur) to sunset (Iftar) (1). The duration of each fasting period may range from 10 to 20 h, depending on the season and geographical location (2). Approximately 463 million adults worldwide have diabetes, and this number is expected to grow by 51% over the next 25 years; it is estimated that 150 million people with diabetes worldwide are Muslim (2, 3). Exemptions are applicable to certain groups, including elderly Muslims, travelers, pregnant or nursing women, and individuals with severe medical conditions such as diabetes. Nonetheless, many Muslims in these groups still choose to fast voluntarily during Ramadan (3).

Given the metabolic characteristics of the disease, individuals with diabetes face an increased risk of complications resulting from significant fluctuations in food and fluid intake. Such complications may encompass health risks including hypoglycemia, hyperglycemia, dehydration, and acute metabolic disturbances such as diabetic ketoacidosis (DKA) (2, 4). To address these risks, the International Diabetes Federation has collaborated with the Diabetes and Ramadan International Alliance (IDF-DAR) to publish detailed guidelines for managing diabetes during Ramadan. These include structured risk assessments before Ramadan and drug-specific recommendations. These guidelines remain the most widely accepted resource for both clinicians and patients (2).

In parallel, the use of artificial intelligence (AI) chatbots has expanded rapidly among the general population, with many people using these tools in everyday information-seeking, and members of the community increasingly use them for health-related questions, often as a first port of call before, or instead of, contacting a healthcare professional that was evident by a survey of the public report that a substantial proportion of adults have already used generative AI for health information (5–7). The appeal is self-evident: chatbots are accessible around the clock, provide instantaneous responses, and support multiple languages, unlike traditional clinic appointments. However, prior evaluations of AI chatbots have shown that ChatGPT is not ready to assume the coaching role for both healthcare learners and healthcare professionals. The inconsistency observed in responses to identical queries presents challenges for learners and decision-makers alike (8). AI chatbot performance also has been shown to differ across languages in answering datasets from medical exams (9). Additionally, the same chatbot might give different responses to repeated identical queries, raising concerns about reproducibility that are seldom measured in published evaluations (10).

Few studies have examined Ramadan-specific medication advice, and to our knowledge none has compared multiple chatbots in both English and Arabic using a guideline-anchored scoring framework. Given that community members may already be using these tools to make real decisions about their medications, an evaluation framed around the patient as the end user is needed. Therefore, the study aims to evaluate the accuracy, completeness, safety, and reproducibility of three leading AI chatbots in providing antidiabetic medication adjustment advice for Ramadan, benchmarked against the IDF-DAR guidelines (2) in English and Arabic. This will provide a basis for determining whether these tools deliver guideline-concordant advice that is sufficiently consistent—across repeated queries and languages—to be considered safe for unsupervised use by patients in the community.

## 2 Methods

### 2.1 Study design

This was a structured, comparative, cross-sectional evaluation of three publicly available AI chatbots—OpenAI ChatGPT, Google Gemini, and Microsoft Copilot—assessed against the IDF-DAR Practical Guidelines for Diabetes Management During Ramadan as the gold standard (Table 1). The protocol used a within-prompt repeated-measures design, in which each prompt was presented to each chatbot in two languages (English and Arabic) and repeated to assess intra-model reproducibility across two periods: day 1 (1 March 2026) and day 14 (15 March 2026).

**Table 1:** Characteristics of the AI chatbots evaluated in this study.

Chatbot	Developer	Version used	Primary functionality	Availability	Notes
ChatGPT	OpenAI (San Francisco, CA, USA)	GPT-5.3	General-purpose large language model capable of natural language understanding, text generation, reasoning, translation, and question answering	Free version with optional paid subscription (Plus)	All chatbot responses were generated via publicly accessible web interfaces in fresh, signed-out browser sessions to minimize carryover effects from prior interactions. Responses were obtained using the default model available to free users at the time of data collection (March 2026)
Gemini	Google (Mountain View, CA, USA)	Flash 3.1	Multimodal large language model supporting conversational question answering, text generation, reasoning, translation, and integration with Google Search when appropriate	Freely accessible with optional premium subscription (Google AI Pro)	
Copilot	Microsoft (Redmond, WA, USA)	Smart mode	AI assistant supporting conversational question answering, text generation, summarization, translation, and web-assisted information retrieval	Freely accessible with optional premium subscription (Copilot Pro)	

### 2.2 Prompts

Twenty-three prompts were designed to reflect the spectrum of antidiabetic regimens commonly prescribed to adults with type 1 and type 2 diabetes. The prompts covered biguanides (metformin); short-acting and modified-release sulfonylureas; meglitinides; dipeptidyl peptidase-4 (DPP-4) inhibitors; SGLT2 inhibitors; glucagon-like peptide-1 (GLP-1) receptor agonists; thiazolidinediones; α-glucosidase inhibitors; basal, rapid-acting, and premixed insulins; basal-bolus regimens; and selected combination regimens. Each prompt followed a standardized template that included patient age, type of diabetes, current medication(s) and dose, and a single direct question about Ramadan adjustment. Prompts were framed as they would plausibly be posed by a patient or family member in the community, rather than by a healthcare professional, and were submitted in the period immediately preceding Ramadan 1447 AH. Each prompt was entered into ChatGPT, Google Gemini, and Microsoft Copilot using a fresh, signed-out session to avoid contamination from prior interactions. Each prompt was queried twice in English and twice in Arabic on independent days (day 1 and day 14), yielding up to four responses per chatbot per prompt. Arabic queries were drafted by a native Arabic speaker to ensure linguistic and clinical equivalence with the English version. The complete English and Arabic prompts are provided in Supplementary Table S1.

### 2.3 Outcomes and scoring

Three primary outcomes were scored for each response. Accuracy (0–2 ordinal scale): 0 = response contradicts the IDF-DAR guideline or contains a clinically harmful recommendation; 1 = response is partially consistent with the guideline; 2 = response is fully consistent with the IDF-DAR recommendation. Completeness (0–4 ordinal scale): the proportion of IDF-DAR–recommended elements addressed in the response, including dose adjustment, timing, glucose monitoring, hypoglycemia counseling, and indications to break the fast. Safety (0–3 ordinal scale): a global rating of the response's patient-safety profile, accounting for the presence of safety warnings, advice to consult a clinician, and the absence of potentially harmful instructions (Table 2). Each chatbot response was assigned a single score at each assessment time point using the predefined scoring rubric. When uncertainty or disagreement arose regarding the appropriate score, the case was discussed among the study investigators until consensus was reached. The evaluator was aware of the chatbot's identity.

**Table 2:** Outcome definitions, assessment criteria, and scoring system.

Outcome	Definition	Assessment criteria	Scoring
Accuracy score	Agreement with the IDF-DAR guideline	Correct dose adjustment; correct timing adjustment	0–2 ordinal scale
Completeness score	Extent to which all recommended counseling components were included	Dose adjustment; timing adjustment; glucose monitoring; advice to break the fast when indicated; hydration advice; pre-Ramadan assessment	0–4 ordinal scale
Safety score	Overall patient-safety profile of the response	Harmful recommendations; omission of critical safety advice; recommendations increasing hypoglycemia or other clinical risks	0–3 ordinal scale

### 2.4 Statistical analysis

The response was the unit of analysis, totaling 276 (calculated from 23 prompts × 3 chatbots × 2 languages × 2 days). Since accuracy (0–2), completeness (0–4), and safety (0–3) were measured on ordinal scales, outcomes were summarized with medians and interquartile ranges (IQRs), as well as means and standard deviations (SDs) for descriptive comparison. The distribution of accuracy categories (fully consistent, partially consistent, and harmful/contradictory) was also shown as frequencies and percentages. The effects of chatbot, language, and their interaction on each outcome were analyzed using generalized linear mixed models (GLMMs) with a multinomial distribution and a cumulative logit link. Prompt (case) was treated as a random intercept to account for the repeated-measures design, where each prompt was assessed by all chatbots in both languages on two occasions. Fixed effects were evaluated via Type III tests.

2-week reproducibility (day 1 vs. day 14) was measured using quadratically weighted Cohen's κ, with 95% confidence intervals and *p*-values to assess significance. Agreement levels were interpreted as poor (< 0.20), fair (0.21–0.40), moderate (0.41–0.60), substantial (0.61–0.80), or almost perfect (0.81–1.00). All tests were two-sided, with *p* < 0.05 indicating statistical significance. Analyses were conducted using IBM SPSS Statistics version 32 (IBM Corp., Armonk, NY, USA).

## 3 Results

### 3.1 Overall performance

**Figure 1:**
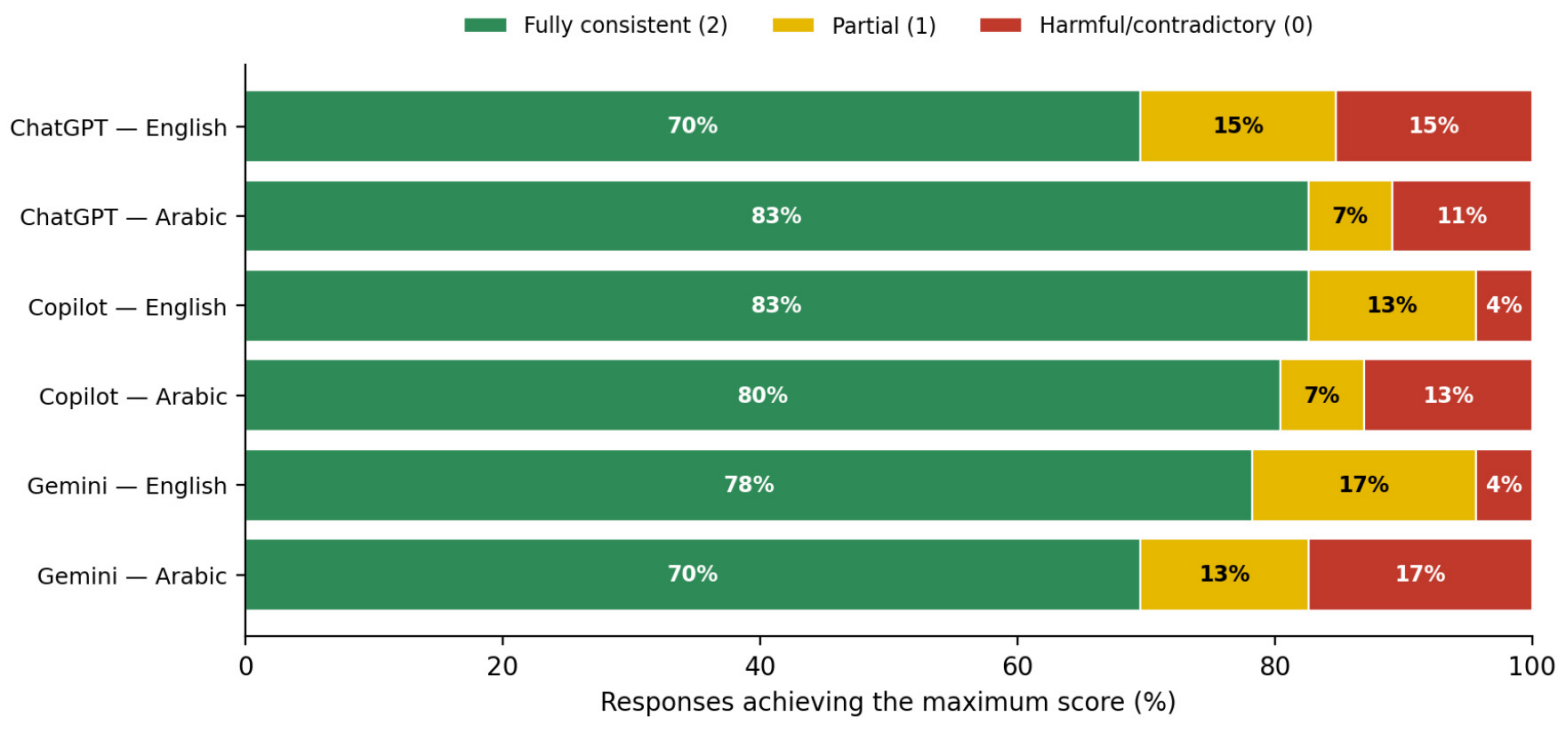
Distribution of accuracy ratings by chatbot and language.

**Figure 2:**
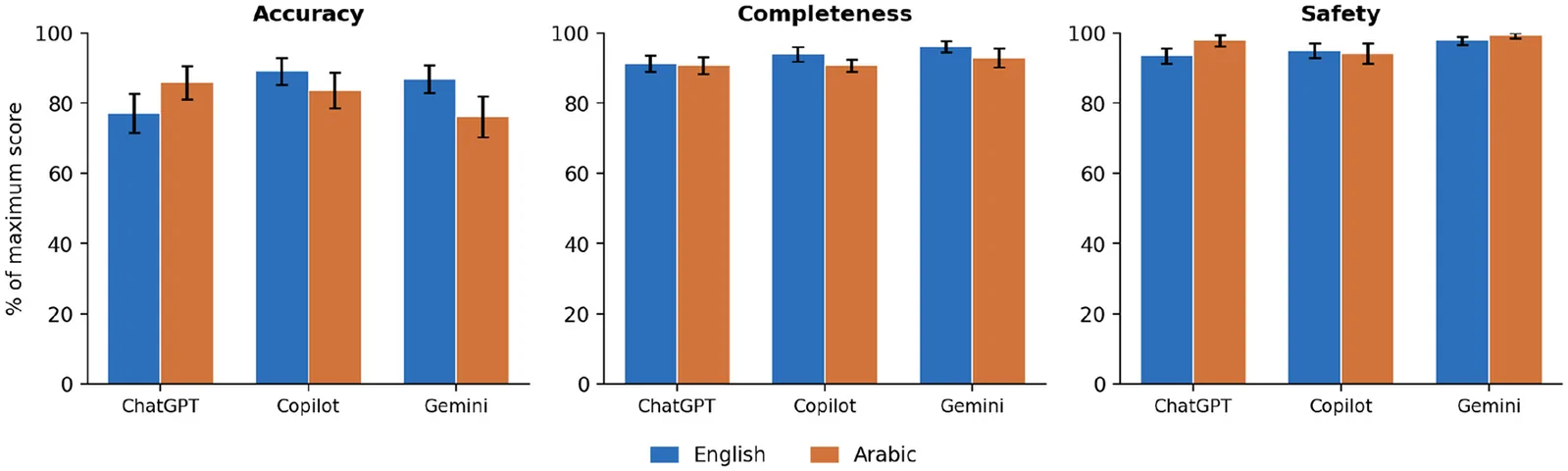
Mean response quality (as a percentage of the maximum attainable score) by chatbot and language.

Out of 276 responses analyzed, 213 (77%) fully followed the IDF-DAR guideline, 33 (12%) were partially compliant, and 30 (11%) either conflicted with the guideline or provided clinically unsafe advice (Figure 1). Overall, the responses showed high completeness and safety: the median completeness score was 4 out of 5 (76% of responses achieved the top score), and the median safety score was 3, with 91% reaching the highest rating. Performance metrics by chatbot and language are shown in Table 3 and Figure 2. ChatGPT had slightly higher average accuracy in Arabic than in English responses (1.72 ± 0.66 vs. 1.54 ± 0.75). Gemini, on the other hand, showed higher accuracy in English than Arabic (1.74 ± 0.53 vs. 1.52 ± 0.78). Copilot's accuracy was similar across languages (1.78 ± 0.51 in English and 1.67 ± 0.70 in Arabic). Completeness scores remained high overall, with Gemini scoring the highest in both English (3.85 ± 0.42) and Arabic (3.72 ± 0.72). ChatGPT and Copilot displayed comparable scores across languages. Safety ratings showed little variation, with all chatbot–language combinations reaching the maximum median score of 3 (IQR, 3–3). Mean safety scores ranged from 2.80 to 2.98, indicating most responses received the highest safety ratings regardless of the chatbot or language used.

**Table 3:** Median (IQR) and mean ± SD of accuracy, completeness, and safety scores by chatbot and language.

Chatbot	Language	Accuracy median (IQR)	Accuracy mean ±SD	Completeness median (IQR)	Completeness mean ±SD	Safety median (IQR)	Safety mean ±SD
ChatGPT	English	2 (1–2)	1.54 ± 0.75	4 (3–4)	3.65 ± 0.60	3 (3–3)	2.80 ± 0.45
	Arabic	2 (2–2)	1.72 ± 0.66	4 (3–4)	3.63 ± 0.64	3 (3–3)	2.93 ± 0.33
Copilot	English	2 (2–2)	1.78 ± 0.51	4 (4–4)	3.76 ± 0.57	3 (3–3)	2.85 ± 0.42
	Arabic	2 (2–2)	1.67 ± 0.70	4 (3–4)	3.63 ± 0.49	3 (3–3)	2.83 ± 0.61
Gemini	English	2 (2–2)	1.74 ± 0.53	4 (4–4)	3.85 ± 0.42	3 (3–3)	2.93 ± 0.25
	Arabic	2 (1–2)	1.52 ± 0.78	4 (4–4)	3.72 ± 0.72	3 (3–3)	2.98 ± 0.15

### 3.2 Differences between chatbots

Generalized linear mixed models found no significant effect of the chatbot on accuracy (F = 0.79, *p* = 0.454), completeness (F = 2.71, *p* = 0.068), or safety (F = 1.76, *p* = 0.175) (Table 4). Query language also did not significantly affect these outcomes, including accuracy (F = 0.07, *p* = 0.791), completeness (F = 2.09, *p* = 0.150), or safety (F = 3.47, *p* = 0.064). Additionally, there was no significant interaction between chatbot and language for accuracy (F = 2.53, *p* = 0.082), completeness (F = 0.82, *p* = 0.440), or safety (F = 0.72, *p* = 0.486), indicating consistent performance of the chatbots across English and Arabic queries. Overall, the three chatbots showed no statistically significant differences in accuracy, completeness, or safety, demonstrating similar performance across all evaluated outcomes.

**Table 4:** Generalized linear mixed models for chatbot, language, and their interaction for each outcome.

Outcome	Effect	F	df	*p*
Accuracy	Chatbot	0.79	2, 269	0.454
	Language	0.07	1, 269	0.791
	Chatbot × language	2.53	2, 269	0.082
Completeness	Chatbot	2.71	2, 268	0.068
	Language	2.09	1, 268	0.150
	Chatbot × language	0.82	2, 268	0.440
Safety	Chatbot	1.76	2, 268	0.175
	Language	3.47	1, 268	0.064
	Chatbot × language	0.72	2, 268	0.486

### 3.3 Effect of language and the chatbot × language interaction

There was no significant main effect of language on any outcome (accuracy F = 0.07, *p* = 0.791; completeness F = 2.09, *p* = 0.150; safety F = 3.47, *p* = 0.064), and no significant omnibus chatbot × language interaction (accuracy, *p* = 0.082; completeness, *p* = 0.440; safety, *p* = 0.486). Overall, 213 of 276 responses (77.2%) fully aligned with the IDF–DAR guidelines, 33 (12.0%) were partially aligned, and 30 (10.9%) contained harmful or guideline-contradictory recommendations. The proportion of fully aligned responses was similar between English and Arabic (76.8% vs. 77.5%), but responses with harmful or contradictory content were more common in Arabic than in English [19/138 (13.8%) vs. 11/138 (8.0%)]. Among individual chatbots, Copilot produced the highest percentage of fully aligned responses in English (82.6%), while ChatGPT led in Arabic with 82.6%. Gemini had the highest rate of harmful or contradictory responses in Arabic (17.4%), and ChatGPT had the highest in English at 15.2%. The accuracy distribution by chatbot and language is detailed in Table 5.

**Table 5:** Distribution of accuracy ratings by chatbot and language.

Chatbot	Language	Fully consistent *n* (%)	Partial *n* (%)	Harmful/contradictory *n* (%)	Total
ChatGPT	English	32 (69.6)	7 (15.2)	7 (15.2)	46
	Arabic	38 (82.6)	3 (6.5)	5 (10.9)	46
Copilot	English	38 (82.6)	6 (13.0)	2 (4.3)	46
	Arabic	37 (80.4)	3 (6.5)	6 (13.0)	46
Gemini	English	36 (78.3)	8 (17.4)	2 (4.3)	46
	Arabic	32 (69.6)	6 (13.0)	8 (17.4)	46
All English		106 (76.8)	21 (15.2)	11 (8.0)	138
All Arabic		107 (77.5)	12 (8.7)	19 (13.8)	138
All responses		213 (77.2)	33 (12.0)	30 (10.9)	276

### 3.4 2-week reproducibility

Reproducibility over 2 weeks was evaluated using quadratically weighted Cohen's κ (see Table 6). Exact agreement between Day 1 and Day 14 was 67.4% for accuracy, 68.1% for completeness, and 83.3% for safety. The quadratic-weighted κ indicated fair reproducibility for accuracy (κ = 0.20) and completeness (κ = 0.29), while safety showed a very low κ (0.02). This low safety κ should be understood in light of the significant ceiling effect, as over 89% of responses received the maximum safety score on both days, creating a highly skewed distribution that tends to lower κ values even when observed agreement is high.

**Table 6:** 2-week reproducibility (day 1 vs. day 14): quadratic-weighted Cohen's κ by outcome.

Outcome	Exact agreement	Weighted κ	95% CI	Z	*p*
Accuracy	67.4%	0.20	0.03 to 0.37	2.60	0.009
Completeness	68.1%	0.29	0.07 to 0.50	3.38	0.001
Safety	83.3%	0.02	−0.15 to 0.18	0.24	0.81

Quadratic weighting κ is interpreted as poor (< 0.20), fair (0.20–0.40), moderate (0.41–0.60), substantial (0.61–0.80), or almost perfect (0.81–1.00).

## 4 Discussion

In this structured evaluation, the three publicly available AI chatbots mostly offered Ramadan medication guidance aligned with the IDF-DAR guideline, with 77.2% of their responses fully consistent. This indicates that general-purpose AI chatbots can often generate advice that aligns with established guidelines for common diabetes medication scenarios during Ramadan. This aligns with previous findings that large language models can answer diabetes-related questions with reasonable accuracy, but their performance is not perfect and is context-dependent (11). In another study, researchers assessed the performance of publicly available AI chatbots in answering medication-related clinical queries. They found that while these chatbots often provided clinically appropriate recommendations, their accuracy and completeness varied, with discrepancies observed across platforms and in repeated queries (12).

Although overall performance was strong, 10.9% of responses were deemed harmful or inconsistent with guidelines. Although this is a minority, such errors could have serious clinical consequences, as patients might follow the chatbot's advice to change medications without consulting healthcare professionals. During Ramadan fasting, incorrect guidance on insulin or glucose-lowering drugs could increase the risk of hypoglycemia, hyperglycemia, dehydration, or other fasting-related issues. Certain features of current large language models may cause these errors. Their probabilistic response generation can lead to inconsistent replies to the same prompt and sometimes produce advice that isn't fully aligned with clinical guidelines. Factors such as prompt wording, the clinical details shared, and variations in training data across languages—especially between English and Arabic—may influence response accuracy. These results highlight that existing AI chatbots should be viewed as supplementary health information sources, not replacements for personalized medical advice, especially when medication adjustments are needed during Ramadan. The IDF-DAR Practical Guidelines highlight the importance of individualized risk assessment, regular glucose monitoring, recognizing hypoglycemia, staying hydrated, and providing clear instructions for breaking the fast (2). Missing or misrepresenting these key points could lead to preventable harm or even fatal outcomes. In a study that included 131 diabetic patients who fasted, 50% were at very high risk, and 3.3% were at high risk, requiring breaking of the fast. However, none of the participants required hospitalization (13).

The generalized linear mixed models revealed no statistically significant effects of chatbot, language, or their interaction on accuracy, completeness, or safety. As a result, the data do not support asserting that any one chatbot outperforms the others. Instead, all three systems demonstrated broadly similar performance across the evaluated metrics. However, the descriptive distribution remains clinically relevant: harmful or contradictory responses were more frequent in Arabic than in English. Although this language difference was not statistically significant in our study, it aligns with broader concerns that LLM performance in healthcare may vary across languages, especially when high-quality medical training data in specialized fields is less abundant outside English. This was evident in a study where English outperformed Arabic across five infectious disease topics. The four AI models achieved an “excellent” rating in English, significantly surpassing their “above-average” Arabic scores (*P* = 0.002) (14).

Although overall completeness scores were high for all three chatbots, our results reveal that responses aligned with guidelines were not always fully comprehensive. While many answers correctly covered medication dose or timing adjustments, some lacked key counseling elements from the IDF-DAR guideline, such as blood glucose monitoring, hydration guidance, indications for breaking the fast during hypoglycemia, and the necessity of a pre-Ramadan risk assessment. These elements are essential for safe diabetes management during Ramadan, and their absence could undermine clinical usefulness and pose serious health risks. A study by Al Hamid et al. assessed the AI chatbots' completeness in responding to diabetes and hypertension prompts. While all chatbots provided accurate information, some information was missing (12). While some of those errors may lead to harm to the patient (8). Additionally, another study evaluated the nutritional advice given by AI chatbots, finding that ChatGPT 4.0 and Claude achieved 77.8%, compared to Copilot's 23.3% (15).

Reproducibility over 2 weeks was limited, with weighted κ values showing fair agreement for accuracy and completeness and negligible agreement for safety. This suggests that the same prompts, given 2 weeks apart, could yield notably different ratings, particularly for accuracy and completeness. Limited reproducibility raises concerns for patient-facing AI tools, as patients expect consistent advice in response to the same questions. Previous studies have also demonstrated that outputs from large language models (LLMs) can vary depending on factors like the model used, prompt structure, and prompt complexity (16).

Safety showed a high exact agreement of 83.3% between Day 1 and Day 14. However, the quadratic-weighted κ was only 0.02. This difference is due to the highly skewed safety ratings, with most responses scoring the maximum at both time points. Such a distribution increases chance agreement, causing the prevalence paradox and resulting in a low κ despite strong actual agreement.

To our knowledge, this is the first research to evaluate the accuracy, completeness, safety, and reproducibility of AI chatbots' advice on antidiabetic medication during Ramadan. Nevertheless, certain limitations should be acknowledged. Firstly, the study examined only three chatbots, whose performance may change with model updates. Secondly, the scoring relied on the investigator's judgment, which may introduce subjectivity despite employing a predefined rubric. Thirdly, the small sample—consisting of 23 prompts and 276 responses—may restrict the generalizability of the results. Finally, ceiling effects concerning safety limited the ability of agreement measures to thoroughly assess reproducibility.

The findings of this study should be interpreted within the specific clinical context examined. The assessment focused on AI-generated advice for diabetes medication management during Ramadan fasting; therefore, the observed performance, safety, and reproducibility may not generalize to other diseases, medical specialties, or clinical decision-making contexts. AI chatbot performance is likely to vary with the clinical task, scenario complexity, and the information provided in the prompt. Further studies across different clinical conditions and healthcare contexts are needed before broader conclusions about the reliability of these tools for patient-directed medical advice can be drawn. Future studies should also encompass a broader range of large language models, including those tailored for medical purposes, premium versions, and subsequent model updates. It should also incorporate real-world patient inquiries and assessments by multidisciplinary experts. The impact of prompt engineering, multilingual capabilities, and retrieval-augmented generation on compliance with guidelines, as well as the potential for harmful recommendations, warrants thorough examination. Given the variability observed across assessments, future studies should evaluate reproducibility by conducting multiple repeated queries at additional time points, rather than relying on two assessments alone, and incorporate complementary measures of response stability. Longitudinal evaluations would also help determine whether reproducibility changes following model updates. Ultimately, embedding evidence-based clinical guidelines into AI systems and evaluating their performance across diverse healthcare settings may foster safer, more dependable AI-supported diabetes management during Ramadan.

## 5 Conclusion

AI chatbots demonstrated comparable performance in providing guideline-based advice for diabetes management during Ramadan, with no notable differences in accuracy, thoroughness, or safety. Most responses followed the IDF-DAR guideline, but some harmful suggestions remained, and response consistency varied over time. These results suggest that while general AI chatbots can offer valuable educational support for patients interested in fasting during Ramadan, they should not substitute for personalized medical advice or clinical judgment. Further improvements in guideline adherence, multilingual performance, and response consistency are needed before AI chatbots can be considered reliable decision-support tools in diabetes care.

## Data Availability

The raw data supporting the conclusions of this article will be made available by the authors, without undue reservation.
