# Supplementary material for: AI Chatbots as a source of Ramadan medication management advice for patients with diabetes: a multilingual comparative evaluation
Source: Front Med (Lausanne). 2026 Aug 28;13:1934432. doi: 10.3389/fmed.2026.1934432 (PMC13584903; doi:10.3389/fmed.2026.1934432)
Supplement: Supplementary file 1 [file Table_1.docx]

# Supplementary Table S1. Standardized English and Arabic prompts used to evaluate AI chatbot responses during Ramadan

| **Case** | **Therapeutic class** | **Clinical scenario** | **English prompt (summary)** | **Arabic prompt (summary)** |
| --- | --- | --- | --- | --- |
| 1 | SGLT2 inhibitor | Empagliflozin 25 mg OD | T2DM on empagliflozin 25 mg once daily. | مريض سكري نوع 2 يتناول إمباغليفلوزين 25 ملغ يوميًا. |
| 2 | Biguanide | Metformin 1000 mg BID | T2DM on metformin 1000 mg twice daily. | مريض سكري نوع 2 يتناول ميتفورمين 1000 ملغ مرتين يوميًا. |
| 3 | Basal insulin | Insulin glargine | T2DM on insulin glargine 16 units at bedtime. | مريض سكري نوع 2 يتناول إنسولين غلارجين 16 وحدة قبل النوم. |
| 4 | DPP-4 inhibitor | Linagliptin | T2DM on linagliptin 5 mg daily. | مريض سكري نوع 2 يتناول ليناغليبتين 5 ملغ يوميًا. |
| 5 | GLP-1 RA | Liraglutide | T2DM on liraglutide 1.8 mg daily. | مريض سكري نوع 2 يتناول ليراغلوتايد 1.8 ملغ يوميًا. |
| 6 | Sulfonylurea | Glipizide | T2DM on glipizide 5 mg daily. | مريض سكري نوع 2 يتناول غليبيزيد 5 ملغ يوميًا. |
| 7 | Oral GLP-1 RA | Oral semaglutide | T2DM on oral semaglutide 7 mg daily. | مريض سكري نوع 2 يتناول سيماغلوتيد الفموي 7 ملغ يوميًا. |
| 8 | SGLT2 inhibitor | Dapagliflozin | T2DM on dapagliflozin 10 mg daily. | مريض سكري نوع 2 يتناول داباغليفلوزين 10 ملغ يوميًا. |
| 9 | Biguanide | Metformin 750 mg BID | T2DM on metformin 750 mg twice daily. | مريض سكري نوع 2 يتناول ميتفورمين 750 ملغ مرتين يوميًا. |
| 10 | Meglitinide | Repaglinide | T2DM on repaglinide TID. | مريض سكري نوع 2 يتناول ريباغلينيد ثلاث مرات يوميًا. |
| 11 | α-Glucosidase inhibitor | Acarbose | T2DM on acarbose TID. | مريض سكري نوع 2 يتناول أكاربوز ثلاث مرات يوميًا. |
| 12 | Basal–bolus insulin | Glargine + Aspart | T1DM on basal–bolus insulin. | مريض سكري نوع 1 على نظام إنسولين قاعدي-بلعي. |
| 13 | Basal–bolus insulin | Detemir + Lispro | T1DM on basal–bolus insulin. | مريض سكري نوع 1 على نظام إنسولين قاعدي-بلعي. |
| 14 | Sulfonylurea | Glimepiride | T2DM on glimepiride. | مريض سكري نوع 2 يتناول غليميبيريد. |
| 15 | Sulfonylurea (MR) | Gliclazide MR | T2DM on gliclazide MR. | مريض سكري نوع 2 يتناول غليكلازيد MR. |
| 16 | GLP-1 RA | Weekly semaglutide | T2DM on weekly semaglutide. | مريض سكري نوع 2 يتناول سيماغلوتيد أسبوعيًا. |
| 17 | Dual GIP/GLP-1 agonist | Tirzepatide | Patient using tirzepatide. | مريض يستخدم مونجارو. |
| 18 | Thiazolidinedione | Pioglitazone | T2DM on pioglitazone. | مريض سكري نوع 2 يتناول بيوغليتازون. |
| 19 | DPP-4 inhibitor | Sitagliptin | T2DM on sitagliptin. | مريض سكري نوع 2 يتناول سيتاغليبتين. |
| 20 | Premixed insulin | 30/20 regimen | T1DM on premixed insulin BID. | مريض سكري نوع 1 على إنسولين مخلوط مرتين يوميًا. |
| 21 | Combination therapy | Metformin + Sitagliptin + Empagliflozin | T2DM on triple oral therapy. | مريض سكري نوع 2 على علاج فموي ثلاثي. |
| 22 | Emergency scenario | Hypoglycaemia before Iftar | T1DM with glucose 55 mg/dL before Iftar. | مريض سكري نوع 1 وسكر الدم 55 ملغ/دل قبل الإفطار. |
| 23 | Combination therapy | Glimepiride + Metformin + Liraglutide | T2DM on combined oral and injectable therapy. | مريض سكري نوع 2 على علاج مركب فموي وحقني. |

All English prompts concluded with the standardized question, "What should I do during Ramadan?" The Arabic prompts ended with the equivalent question, "ما الذي يجب عليّ فعله خلال شهر رمضان؟".
